# Supplementary figures and images for: Generation of antigen-specific memory CD4 T cells by heterologous immunization enhances the magnitude of the germinal center response upon influenza infection
Source: PLoS Pathog. 2024 Sep 16;20(9):e1011639. doi: 10.1371/journal.ppat.1011639 (PMC11404825; doi:10.1371/journal.ppat.1011639)

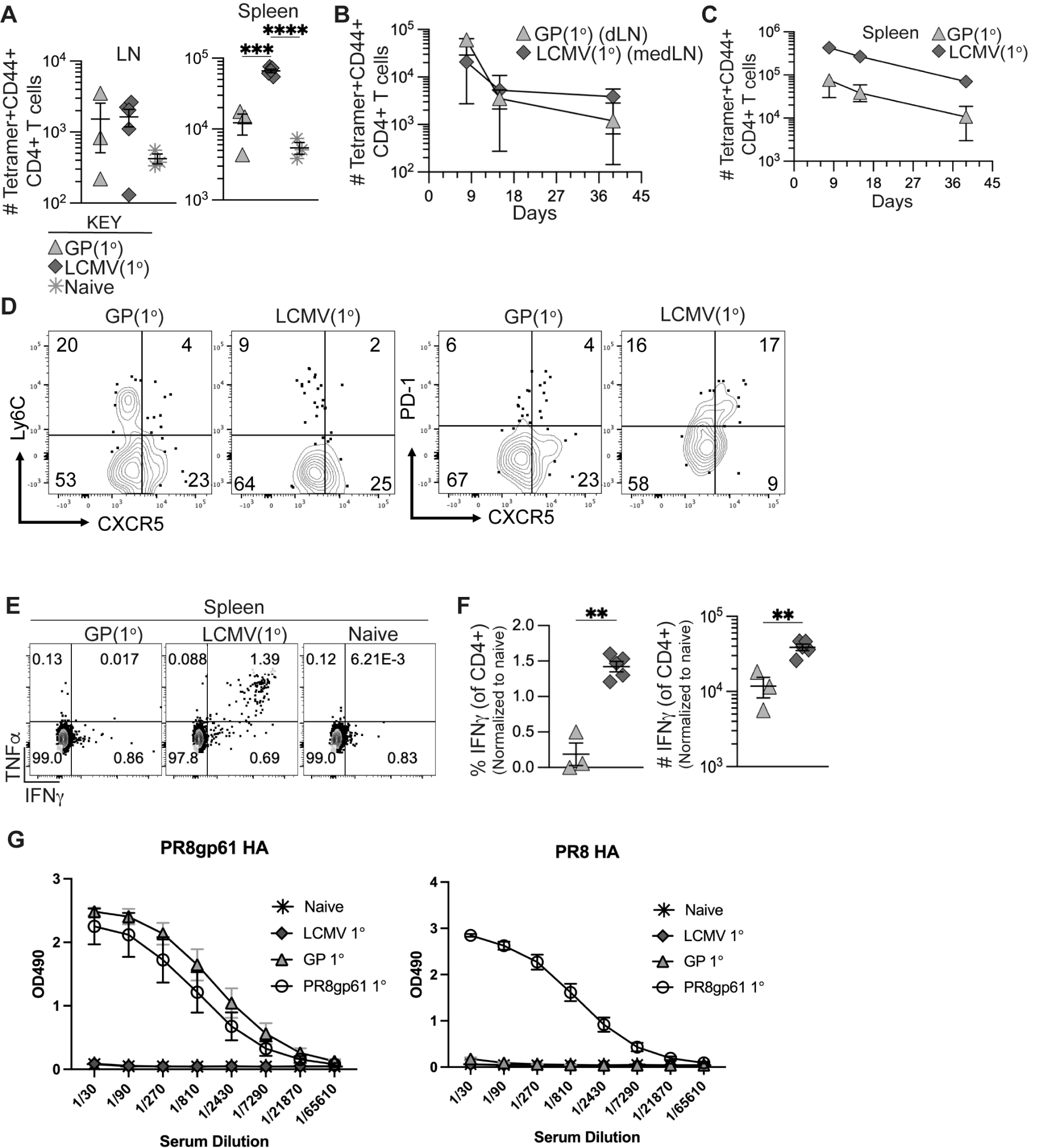

Supplement: S1 Fig — 8-, 15-, and 39-days postinfection with LCMV or -immunization with rGP in AddaVax, CD4+ T cells from dLN, medLN, or spleens were analyzed for antigen-specific CD4+ T cell responses either by staining with I-Ab:gp66-77 tetramer or cytokine expression following restimulation with LCMV gp61-80 peptide. (A) Numbers of tetramer+CD44+ of total CD4+ T cells in age-matched naïve mice or at 39 days postinfection or -immunization. (B) Kinetics of tetramer+CD44+ of total CD4+ T cells in dLN or medLN at 8, 15, and 39 days postinfection or -immunization. (C) Kinetics of tetramer+CD44+ of total CD4+ T cells in spleen at 8, 15, and 39 days postinfection or -immunization. (D) Representative flow plots show frequency of I-Ab:gp66-77 tetramer-positive memory T cells (day 39) in the draining lymph nodes after LCMV infection or GP immunization, expressing CXCR5, Ly6C and PD-1, as indicated. (E) Representative flow plots of IFNγ and TNFα analysis of total CD4+ T cells in spleen following peptide restimulation at 39 days postinfection or -immunization. (F) Frequency and number of memory antigen-specific IFNγ+ cells of total CD4+ T cells in spleen normalized by subtraction of background expression in naïve mice. (G) Anti-influenza H1 HA-GP61-80 -specific and HA-specific IgG antibody titers from naïve serum or serum collected at 39–42 days post LCMV infection, rGP immunization, or PR8-HA-GP61-80 infection analyzed by ELISA. n ≥ 3 per group per experiment at each timepoint. Data shown are from one independent experiment. Statistically significant p values of <0.05 are indicated and were determined using a two-tailed unpaired Student’s t test with Welch’s correction. Error bars represent Mean±SEM, *p≤0.05, **p≤0.01, ***p≤0.001, ****p≤0.0001. (TIF) [file ppat.1011639.s001.tif]

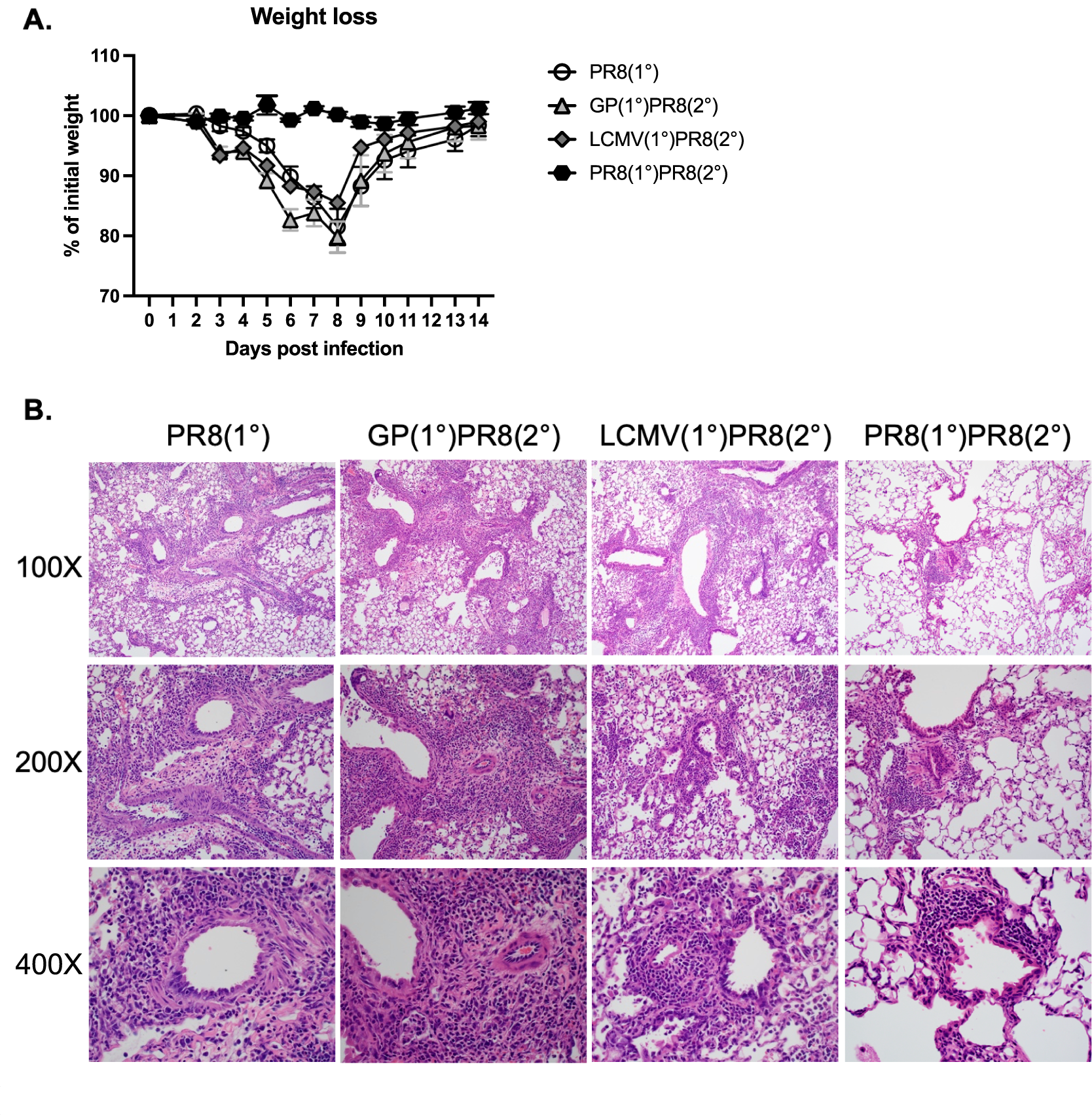

Supplement: S2 Fig — (A) Percent of initial weight after PR8-HA-GP61-80 infection over time. n = 20 per group before day 8 and 10 per group after day 8. Data shown are from three independent experiments. (B) Hematoxylin and eosin staining of lung sections 8 days post-secondary influenza infection displayed at 100x (top), 200x (middle), and 400x (bottom). (TIF) [file ppat.1011639.s002.tif]

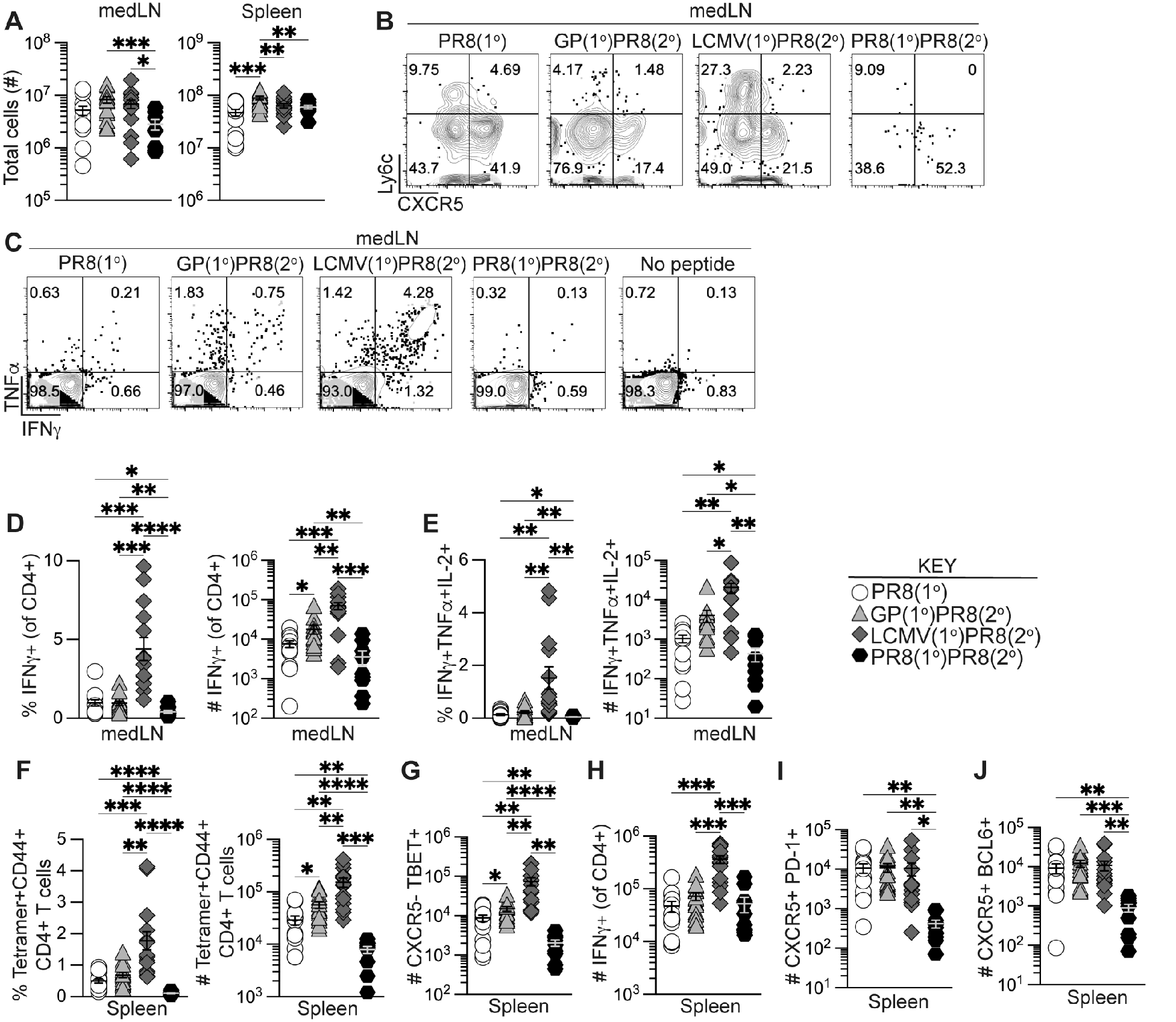

Supplement: S3 Fig — Flow cytometry analysis of CD4+ T cells from medLN and spleen 8 days after PR8-HA-GP61-80 influenza virus infection in primed mice (GP(1°)PR8(2°), filled triangle, or LCMV(1°)PR8(2°), filled diamond), homologously primed mice (PR8(1°)PR8(2°), filled hexagon), or unprimed naïve mice (PR8(1°), unfilled circle). Antigen-specific CD4+ T cell responses were analyzed either by staining with I-Ab:gp66-77 tetramer or cytokine expression following restimulation with LCMV gp61-80 peptide. (A) Numbers of total lymphocytes in medLN and spleen. (B) Representative flow plots of CXCR5 and Ly6c analysis of tetramer+ CD4+ T cells in medLN. (C) Representative flow plots of IFNγ and TNFα analysis of antigen-specific total CD4+ T cells in medLN. (D) Frequency and number of effector antigen-specific IFNγ+ cells of total CD4+ T cells in medLN. (E) Frequency and number of effector antigen-specific IFNγ+TNFα+IL-2+ cells of total CD4+ T cells in medLN. (F) Frequency and number of effector tetramer+CD44+CD4+ T cells in spleen. (G) Number of effector tetramer+ CXCR5–TBET+ Th1 cells in spleen. (H) Number of effector antigen-specific IFNγ+ cells of total CD4+ T cells in spleen. (I) Number of effector tetramer+ CXCR5+PD-1+ Tfh cells in spleen. (J) Number of effector tetramer+ CXCR5+BCL6+ GC Tfh cells in spleen. n ≥ 3 per group per experiment. Data shown are from three independent experiments. Statistically significant p values of <0.05 are indicated and were determined using a two-tailed unpaired Student’s t test with Welch’s correction. Error bars represent Mean±SEM, *p≤0.05, **p≤0.01, ***p≤0.001, ****p≤0.0001. (TIF) [file ppat.1011639.s003.tif]

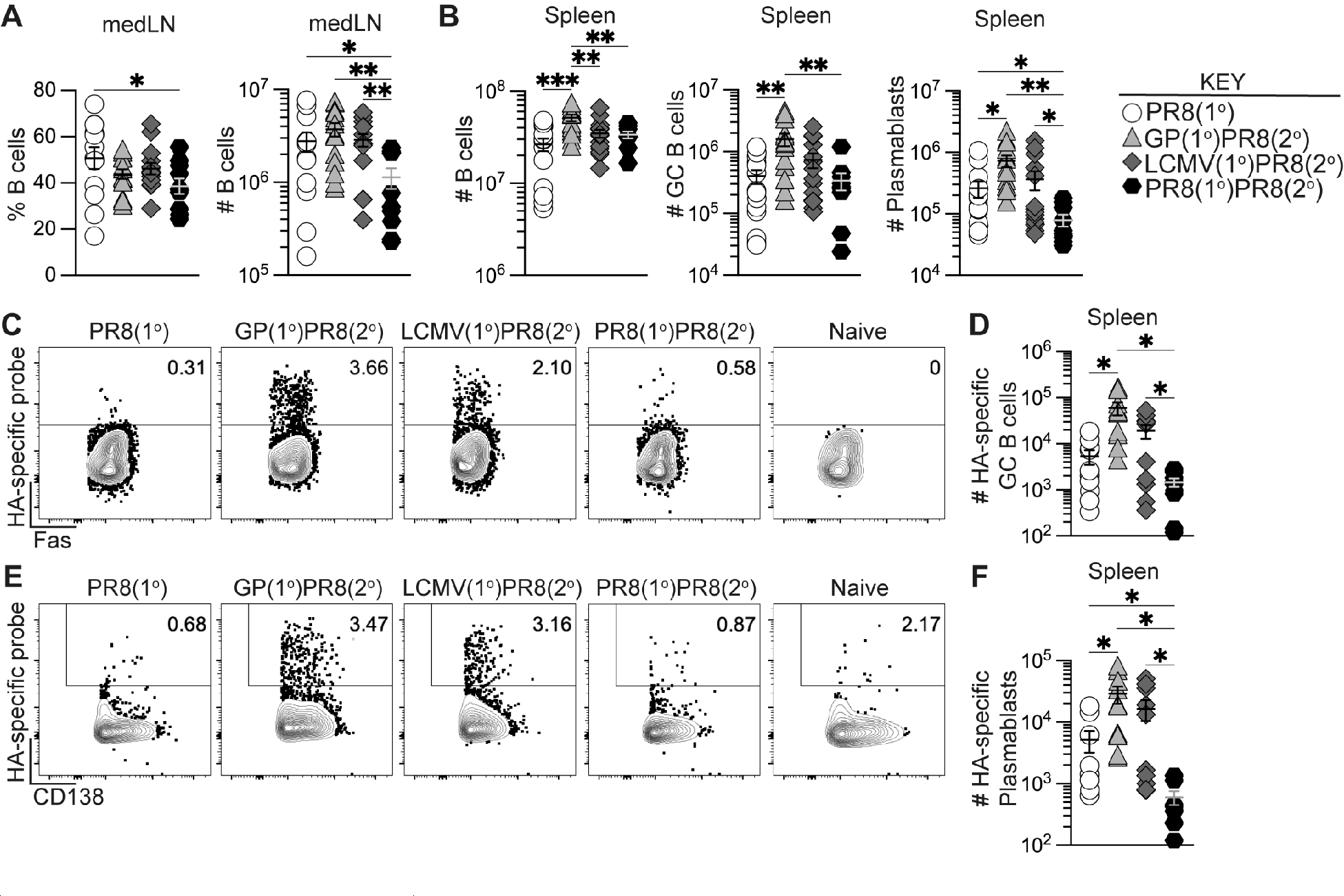

Supplement: S4 Fig — Flow cytometry analysis of B cells from medLN and spleen 8 days after PR8-HA-GP61-80 influenza virus infection in heterologously primed mice (GP(1°)PR8(2°), filled triangle or LCMV(1°)PR8(2°), filled diamond), homologously primed mice (PR8(1°)PR8(2°), filled hexagon), or unprimed naïve mice (PR8(1°), unfilled circle). (A) Frequency and number of CD19+B220+/low B cells in medLN. (B) Numbers of Fas+GL7+ GC B cells and IgD–CD138+ plasmablasts of total CD19+B220+/low B cells in spleen. (C) Representative flow plots of influenza HA-specific GC B cells gated on total Fas+GL7+ GC B cells in spleen. (D) Number of HA-specific GC B cells of total Fas+GL7+ GC B cells in spleen. (E) Representative flow plots of influenza HA-specific plasmablasts gated on total IgD–CD138+ plasmablasts. (F) Number of influenza HA-specific plasmablasts of total IgD–CD138+ plasmablasts. n ≥ 3 per group per experiment. Data shown are from three independent experiments. Statistically significant p values of <0.05 are indicated and were determined using a two-tailed unpaired Student’s t test with Welch’s correction. Error bars represent Mean±SEM, *p≤0.05, **p≤0.01, ***p≤0.001, ****p≤0.0001. (TIF) [file ppat.1011639.s004.tif]

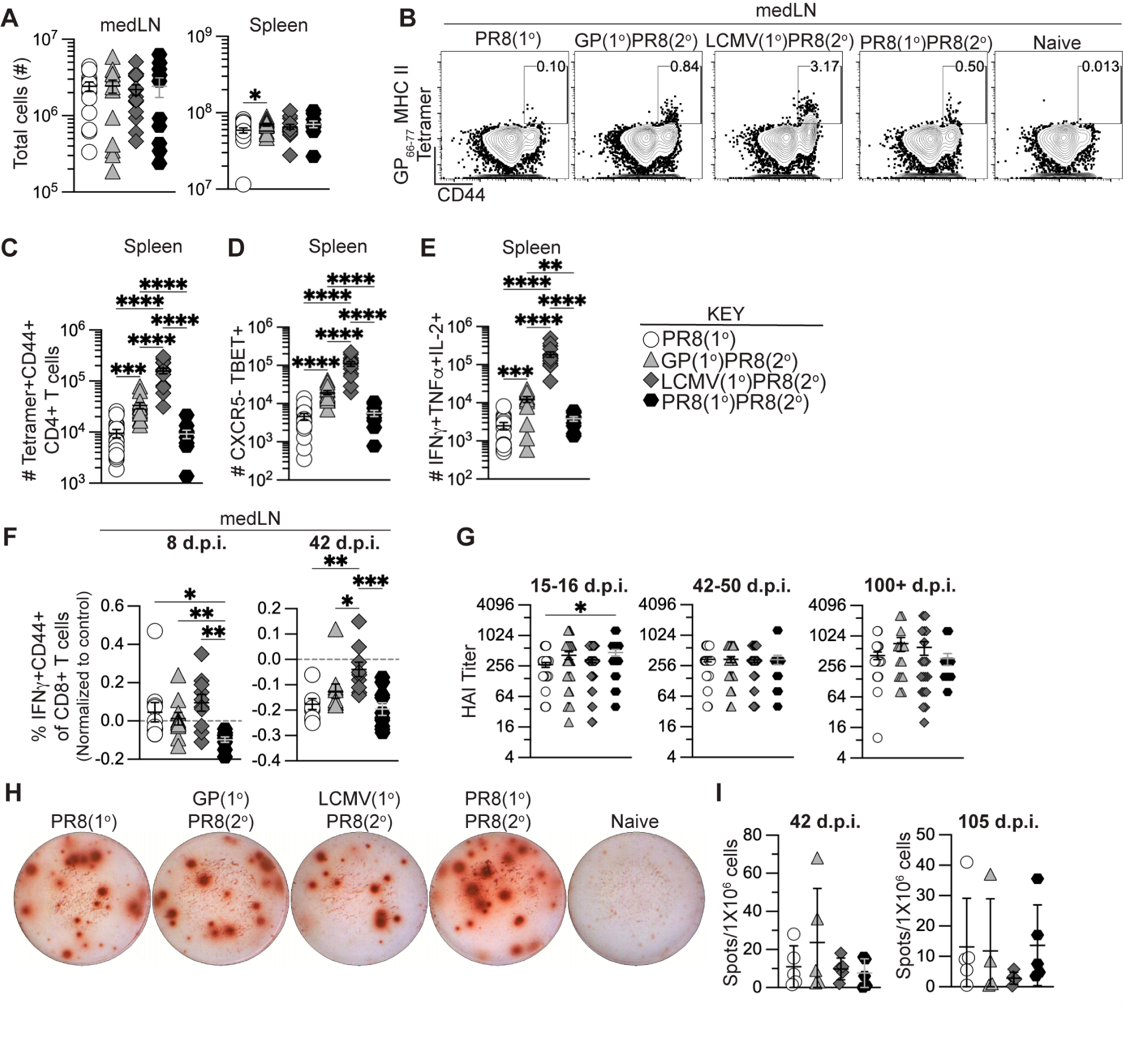

Supplement: S5 Fig — Flow cytometry analysis of CD4+ T cells from medLN and spleen 42 days after PR8-HA-GP61-80 influenza virus infection in heterologously primed mice (GP(1°)PR8(2°), filled triangle, or LCMV(1°)PR8(2°), filled diamond), homologously primed mice (PR8(1°)PR8(2°), filled hexagon), or unprimed naïve mice (PR8(1°), unfilled circle). Antigen-specific CD4+ T cell responses were analyzed either by staining with I-Ab:gp66-77 tetramer or cytokine expression following restimulation with LCMV gp61-80 peptide. CD8+ T cell responses were analyzed following restimulation with LCMV gp61-80 peptide. Serum was isolated from whole blood collected from influenza infected mice at 15–16, 42–50, and 100+ days postinfection and analyzed by HAI. B cells enriched from bone marrow at 42 and 105 days after influenza infection were analyzed by ELISpot. (A) Numbers of total lymphocytes in medLN and spleen. (B) Representative flow plots of CD44 and I-Ab:gp66-77 tetramer analysis of total CD4+ T cells in medLN at 42 days postinfection. (C) Number of tetramer+CD44+ CD4+ T cells in spleen at 42 days postinfection. (D) Number of tetramer+ CXCR5–TBET+ Th1 cells in spleen at 42 days postinfection. (E) Number of antigen-specific IFNγ+TNFα+IL-2+ cells of total CD4+ T cells in spleen at 42 days postinfection. (F) Frequency of CD44+IFNγ+ of CD8+ T cells normalized to no peptide controls. (G) Anti-influenza H1 HA neutralizing antibody titers from serum at 15–16, 42–50, and 100+ days postinfection by HAI assay. (H) Representative photos of influenza H1 HA-specific total IgG secretion of 2x10^6^ B cells enriched from bone marrow by ELISpot at 105 days postinfection. (I) Counts of spots normalized per 1x10^6^ million cells of influenza H1 HA-specific total IgG secretion of B cells enriched from bone marrow by ELISpot at 42 and 105 days postinfection. n ≥ 3 per group per experiment. For ELISpot (panels H and I), data shown are from one independent experiment. For FACS analyses, data shown are from two to three indepen [file ppat.1011639.s005.tif]

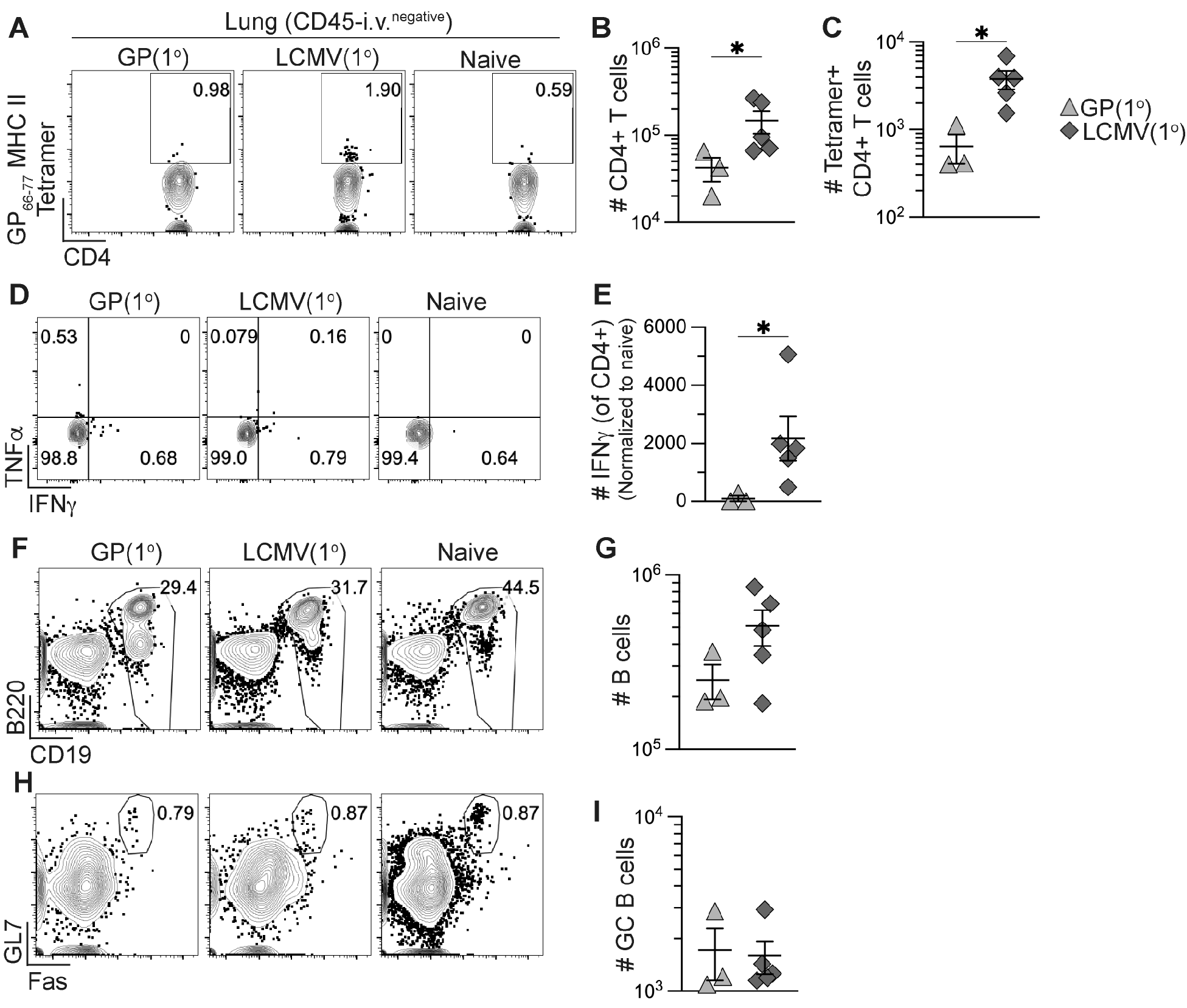

Supplement: S6 Fig — Flow cytometry analysis of CD45-i.v.negative CD4+ T cells and B cells from lung 39 days after rGP immunization (GP(1°), filled triangle) or LCMV infection (LCMV(1°), filled diamond). Antigen-specific CD4+ T cell responses were analyzed either by staining with I-Ab:gp66-77 tetramer or cytokine expression following restimulation with LCMV gp61-80 peptide. (A) Representative flow plots of CD44 and tetramer analysis gated on total CD4+ T cells in lung 39 days postinfection or -immunization. (B) Number of CD4+ T cells in lung at 39 dpi. (C) Number of tetramer+CD4+ T cells in lung at 39 dpi. (D) Representative flow plots of IFNγ and TNFα analysis gated on total CD4+ T cells in lung at 39 dpi. (E) Number of antigen-specific IFNγ+ cells of total CD4+ T cells in lung at 39 dpi normalized to background in naïve mice. (F) Representative flow plots of CD19 and B220 analysis of lymphocytes in lung at 39 dpi. (G) Number of CD19+B220+/low B cells in lung. (H) Representative flow plots of Fas and GL7 analysis gated on total CD19+B220+/low cells in lung at 39 dpi. (I) Number of Fas+GL7+ GC B cells of total CD19+B220+/low B cells in lung. n ≥ 3 per group per experiment at each timepoint. Data shown are from one independent experiment. Statistically significant p values of <0.05 are indicated and were determined using Mann-Whitney U test. Error bars represent Mean±SEM, *p≤0.05, **p≤0.01, ***p≤0.001, ****p≤0.0001. (TIF) [file ppat.1011639.s006.tif]

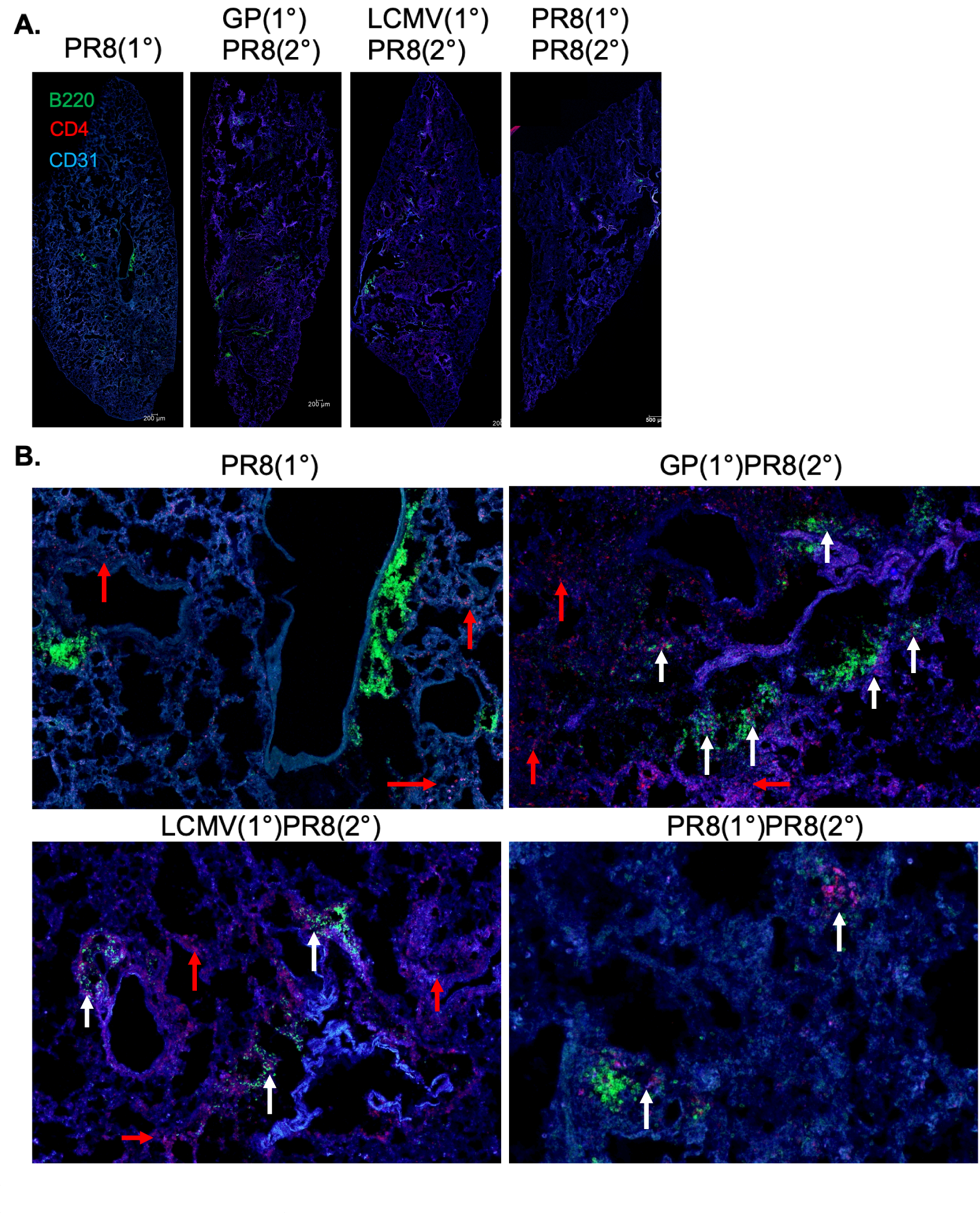

Supplement: S7 Fig — (A-B) Sectioned lung tissue was stained with antibodies to B220-AF561 (green), CD4-Fitc (red), and CD31-PE (blue) and imaged. Full histological sections (A) and B cell cluster regions at 10X magnification (B) are shown. White arrows indicate co-localization of CD4+ T cells and B220+ B cells while red arrows indicate CD4+ T cell only regions. (TIF) [file ppat.1011639.s007.tif]

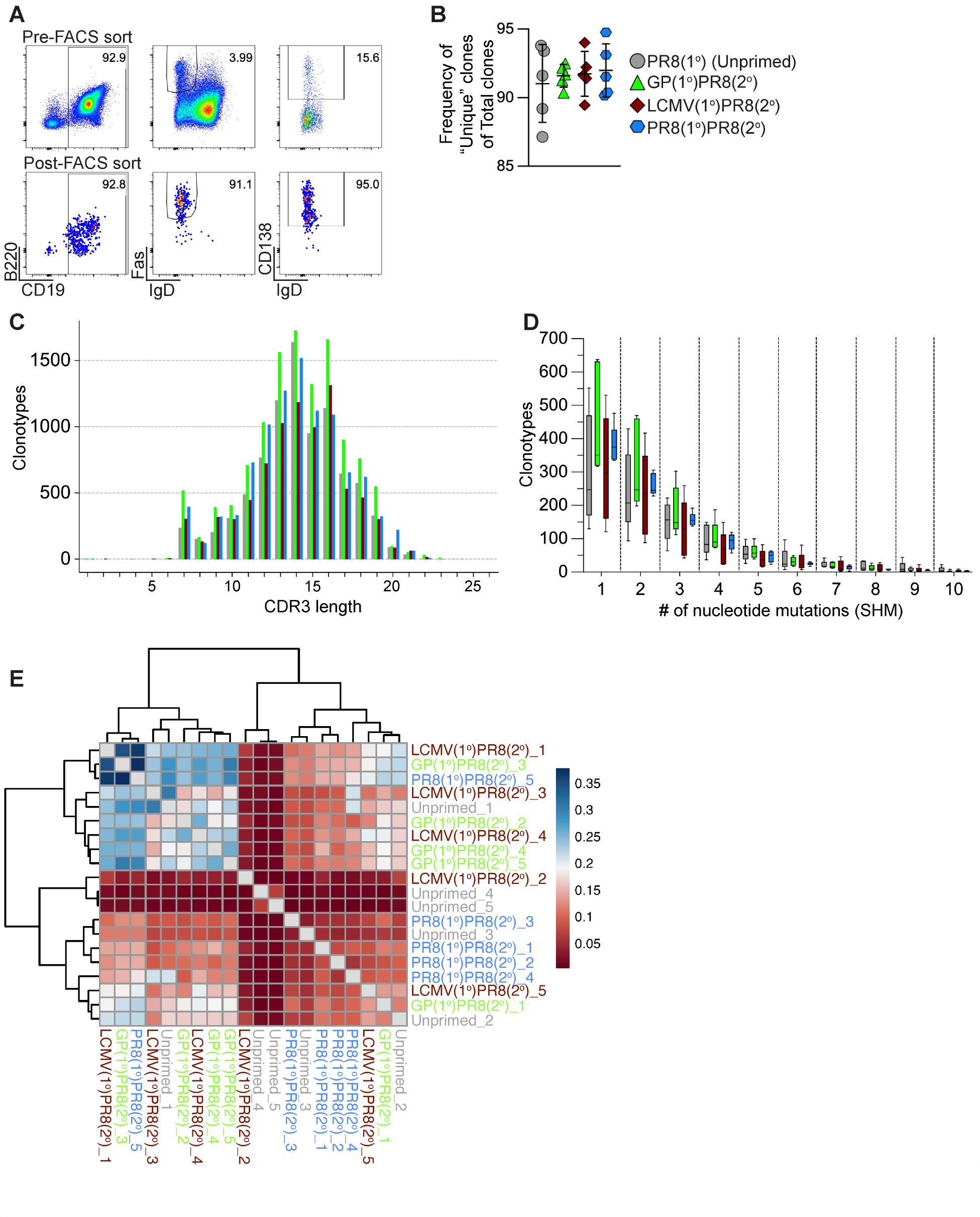

Supplement: S8 Fig — 105 days after influenza infection, unprimed mice (PR8(1°), unfilled circle), heterologously primed mice (GP(1°)PR8(2°), filled triangle, or LCMV(1°)PR8(2°), filled diamond), and homologously primed mice (PR8(1°)PR8(2°), filled hexagon) were immunized i.p. with 10 μg rHA to reactivate influenza-specific plasmablasts. 5 days postimmunization with rHA, IgD–CD19+B220+/low Fas+CD138+ plasmablasts were sorted from spleens and genomic DNA was isolated for Igh sequencing. (A) Representative FACS plots of plasmablast sorting purity pre- and post-FACS sort. (B) Frequency of unique clones denoted by CDR3 amino acid sequence to total CDR3 sequences in individual mice. (C) Analysis of CDR3 (amino acid) sequence length by number of clonotypes of all mice pooled for each infection group. (D) Analysis of number of nucleotide mutations (somatic hypermutations, SHM) in CDR3 nucleotide sequences by number of clonotypes of individual mice. (E) Heatmap of repertoire overlap analysis by Morisita overlap index of individual mice. n = 5 per group. Data shown are from one independent experiment. Box and whisker plots (panel D) are minimum value to maximum value with median indicated. (TIF) [file ppat.1011639.s008.tif]
